# Effect of Dietary Amaranth (*Amaranthus hybridus chlorostachys*) Supplemented with Enzyme Blend on Egg Quality, Serum Biochemistry and Antioxidant Status in Laying Hens

**DOI:** 10.3390/antiox12020456

**Published:** 2023-02-10

**Authors:** Hossein Janmohammadi, Babak Hosseintabar-Ghasemabad, Majid Oliyai, Sadegh Alijani, Ivan Fedorovich Gorlov, Marina Ivanovna Slozhenkina, Aleksandr Anatolievich Mosolov, Lourdes Suarez Ramirez, Alireza Seidavi, Vito Laudadio, Vincenzo Tufarelli, Marco Ragni

**Affiliations:** 1 Department of Animal Science, Faculty of Agriculture, University of Tabriz, Tabriz 51666-16471, Iran; 2 Volga Region Research Institute of Manufacture and Processing of Meat-and-Milk Production, 400131 Volgograd, Russia; 3 Department of Animal Pathology, Animal Production, Bromatology and Food Technology, Veterinary Faculty, University of Las Palmas de Gran Canaria, 35412 Arucas, Spain; 4 Department of Animal Science, Rasht Branch, Islamic Azad University, Rasht 41335-3516, Iran; 5 Department of Precision and Regenerative Medicine and Jonian Area, University of Bari ‘Aldo Moro’, Valenzano, 70010 Bari, Italy; 6 Department of Soil, Plant and Food Sciences, University of Bari ‘Aldo Moro’, 70126 Bari, Italy

**Keywords:** amaranth, enzyme blend, antioxidant status, laying hen, egg quality

## Abstract

A feeding trial was performed to assess the effects of dietary raw amaranth (*Amaranthus hybridus chlorostachys*) grain (RAG), with or without an enzyme blend, on the productive performance, blood biochemistry, and antioxidant status in laying hens. The trial was conducted following a completely randomized design by factorial method, including five levels of RAG (0, 10, 20, 30, and 40%, respectively) and two levels of enzyme blend (0 ^−E^ and 0.025 ^+E^ %). A total of 960 White Leghorn (Hy-line W-36) laying hens (56 weeks of age) were divided into 10 groups with eight repetitions, including 12 birds. The trial period was ten weeks. Results showed that RAG levels in feed (>10%) led to a significant decrease in blood total cholesterol (TC), but they also significantly decreased feed conversion ratio (FCR) (*p* ˂ 0.05) as measured by feed intake (FI), hen daily production (HDP), egg weight (EW), and mass (EM), leading to overall worse productivity compared to the control group. On the contrary, the addition of the enzyme blend led to an improvement in the investigated production traits (*p* ˂ 0.05), with the exception of HDP. The enzyme blend was also capable of recovering productive performance when combined with low concentrations of RAG (10%) (*p* ˂ 0.05), and RAG × enzyme blend groups showed the lowest values of TC (*p* ˂ 0.05). Moreover, the interaction effects for atherogenic index (LDL/HDL) indicated a significant and promising reduction in response to the addition of RAG both in the presence and absence of the enzyme blend (*p* ˂ 0.05), and this additive also significantly reduced levels of egg yolk cholesterol (*p* ˂ 0.05). In summary, the evidence gathered in this trial showed that dietary RAG had positive effects on egg quality characteristics, leading to the production of low-cholesterol eggs, and, at the same time, it may improve the health status of laying hens. Furthermore, the addition of an enzyme blend allowed feeding up to 10% RAG in the diet, leading to an optimal balance between animal productivity and the beneficial effects of RAG.

## 1. Introduction

Food safety is a crucial component of the national security of countries, and the increasing demand for healthy nutrition requires innovative solutions encompassing the whole agri-food sector. In the poultry sector, recent research has established that the quality of meat and eggs for human consumption can be improved, for example, with novel feed formulations that may include supplements with beneficial effects for animal health and productivity [1,2,3]. The use of alternative cereals such as amaranth (*Amaranthus hybridus chlorostachys*) is an option for innovative poultry feeding, allowing to maintain performance and improving animal health, ultimately leading to the production of healthier products such as eggs and meat with low cholesterol [4,5]. A recent report by WHO [6] suggested that the use of amaranth, which is classified among the Neglected and Underutilized Species (NUS), could be a sustainable and low-cost solution for agriculture, with many applications in the food and pharmaceutical industries [7,8].

The genus *Amaranthus* comprises 75 species of dicotyledonous pseudo-cereals in group C4, 11 of which grow in Iran [9,10,11] (Gamel et al., 2007, Ghahremaninejad and Hoseini, 2015; Mozaffariam, 2020). Amaranth has a similar energy content to common cereals, but it is also very rich in other nutrients, especially essential amino acids, making it very advantageous for human and animal diets [7,9,12,13,14]. The presence of bioactive compounds in grains, such as squalene, tocopherols, and phytosterols, makes this plant useful as poultry feed [12,15,16,17,18]. Many studies on the nutritional value of amaranth in human and animal nutrition have been reported since the 1980s, and its use has increased in the last decade [19].

The inclusion of raw, autoclaved, pelleted, and extruded amaranth grains in the diet of broilers, laying hens, quails, and turkeys, and the evaluation of production performance and other health-related parameters indicated the potential use in poultry nutrition [20,21,22,23,24,25,26]. Moreover, a review of the literature showed that the use of amaranth determined a reduction in blood fats and cholesterol in mice, hamsters, rabbits, as well as in poultry [27,28,29,30,31]. Additionally, Punita and Chaturvedi [32] reported that the use of amaranth (raw or processed) at 25% in the diet of laying hens led to the production of eggs with low triglycerides and cholesterol, and with higher levels of linoleic acid. Recently, Rodriguez-Rios et al. [26] studied the effect of dietary amaranth in laying hens and concluded that concentrations up to 15% did not affect production parameters. However, due to the presence of some compounds such as saponins, tannins, phytic acid, oxalate, protease inhibitors, nitrates, and phytohemagglutinins in amaranth, it is necessary to use exogenous enzymes to reduce their negative effects on bird performance and health [33,34,35,36].

The available literature showed that the use of enzymes in poultry nutrition not only improved feed efficiency, but also allowed nutritionists to use unconventional feeds, which are cheaper and more available [37]. Indeed, the use of enzyme blend in poultry feed can improve the digestibility of nutrients, and can support the performance of laying hens [38,39]. Recently, Hosseintabar-Ghasemabad et al. [40] showed that feeding up to 20% of processed amaranth with enzymes in laying hens led to an improvement in egg mass and weight and feed efficiency, while also reducing egg yolk cholesterol. In addition, bird health parameters, including blood lipid profile and antioxidant status, were improved by feeding amaranth with enzymes. The same authors provided information on using raw amaranth, which is abundant in many countries; however, the available literature on its use in laying hens is still scarce.

Building upon the successful and promising experiences of using enzyme additives in cereals, the present study aimed to evaluate the effects of raw amaranth grains with or without enzyme blend in laying hen diets. Furthermore, the bioactive compounds content of raw amaranth grains and the effects of various dietary inclusion levels on hens’ productive performance, blood parameters, and antioxidant status, as well as on egg quality, were assessed.

## 2. Material and Methods

### 2.1. Animals and Diets

All the procedures followed in present research aimed to minimize pain or discomfort of the laying hens and were confirmed by the Ethic, Care, and Use Committee of Animals of the Tabriz University. A total of 960 Hy-Line W-36 White Leghorns (56 weeks of age) were assigned to ten dietary treatments with eight replicates having twelve hens in three separate cages of (41 × 23 × 43 cm) with four birds per cage, in a 5 × 2 factorial arrangement by completely randomized design. The dietary treatments consisted of five levels of raw amaranth grain (RAG; 0, 10, 20, 30, and 40%, respectively) with or without enzyme blend addition (0 and 0.025%, respectively), as reported in Table 1. Before starting the feeding trial, egg production of birds was recorded for each individual, and laying hens with equal egg production were allotted in each replication. Hen body weight (BW) and mortality rate were recorded during the 10 weeks of trial duration. Diets were formulated on the basis of linear programming using the UFFDA (User-friendly Feed Formulation, Done Again) software and according to the catalog of Leghorn hens’ requirements (Table 2). Feed and water were offered ad libitum, and lighting program and temperature rearing conditions followed the recommendations of the Hy-line W-36 White Leghorn laying hens.

### 2.2. Test Ingredients

Raw amaranth (*Amaranthus hybridus chlorostachys*) grains were supplied by Darvash Giah Khazar medicinal herbs complex company (Ltf) (Gilan, Rasht, Iran), after a preliminary analysis of grain chemical composition as reported in our previous studies [8,41] (Hosseintabar-Ghasmabad et al., 2020, Janmohammadi et al., 2022). In order to complete the nutritional analyses of amaranth, in the present study, squalene (method IOC, 2011) and phytosterols [42,43] (Takatsuto and Abe, 1992; Bhandari et al., 2012) were determined via gas chromatography (model 6100, Younglin, Republic of Korea), while tocopherols were evaluated by RP-HPLC (Younglin Acme 9000 model, Republic of Korea) and a dual-channel fluorescence detector (Jasco FP-4025 model, Japan), as reported in Table 3. The enzyme blend used in this trial was Natuzyme P50 (Bioproton Pty Ltf., Sunny bank, QLD, Australia). Enzyme constituents and their respective activity (U/g) per kilogram of diet were as follows: xylanase 10^7^, cellulase 5 × 10^6^, pectinase 5 × 10^4^, and β-glucanase 10^6^, which is of *Trichoderma reesei* of fungal origin, and it included *Trichoderma longibachiatum* and α-amylase from *Bacillus subtilis*, and protease 6 × 10^6^ and phytase 5 × 10^5^ from *Aspergillus niger*.

### 2.3. Sampling and Analyses

During the feeding trial, daily feed intake (FI), egg weight (EW), egg mass (EM), hen-day production (HDP), feed conversion ratio (FCR), and Haugh unit were measured. The FI was assessed daily during the trial period, and weekly feed consumption was calculated by subtracting left-over feed from the quantity supplied to laying hens. Separately, the produced eggs were collected and counted daily from each replicate, and EW was recorded. HDP was calculated as total number of eggs collected divided by total number of live hens per day in each group. EM was calculated as per hen per day by multiplying HDP by EW. At the end of the feeding trial, the FCR was calculated as FI/EM.

In order to evaluate the egg quality traits (shell thickness, shell strength, shape index), nine eggs from each replicate were collected for a total of 720 eggs and assessed 24 h after egg collection. Shell strength was measured by a specific instrument (Digital Egg shell force Gauge; Wagner Instruments, Bridgeport, CT, USA). Shell thickness was measured at three locations (air cell, equator, and sharp end) with a digital micrometer instrument (Mitutoyo, Japan). Yolk diameter (D) of all eggs was measured using a compass (Model: Swordfish, Tokyo, Japan), and height of yolk (HY) was measured using a tripod digital micrometer instrument (Model: Mituoyo, Kawasaki, Japan). Yolk index was calculated as [HY/D] ×100, and Haugh unit was calculated as 100 log HA + 7.57 − 1.7 EW ^0.37^, where the HA, HY, and EW were albumen height, yolk height, and egg weight, respectively. Moreover, to determine yolk cholesterol content, at the end of the trial period, six eggs from each replicate, for a total of 480 eggs, were transferred to lab and stored at −80 °C for further analysis, as described by Baghban-Kanani et al. [34], after separating the yolk from the albumen. At the end of the trial, six hens from each replicate were randomly selected to collect blood samples from the wing vena. Blood samples were stored in additive-free blood tubes, transferred to lab, and then centrifuged for 10 min at 3000 rpm at 20 °C to obtain plasma for analysis. Plasma samples were used to determine triglycerides (TGs), total cholesterol (TC), low-density lipoprotein (LDL), high-density lipoprotein (HDL), alanine aminotransferase (ALT), aspartate aminotransferase (AST), malondialdehyde (MDA), and total antioxidant capacity (TAC) using commercial diagnostic kits (enzymatic method) [44]. All samples were subsequently stored at −85 °C in lab for further analysis. TGs were measured using colorimetric enzyme procedure, and HDL and TC were measured using enzymatic photometric method [45,46]. The atherogenic index was calculated as the LDL/HDL ratio. MDA and TAC activities were determined in plasma samples using RANDOX kits (Germany), according to manufacturer instructions. Plasma lipid peroxidation (LP) was determined according to Kei et al. [47] method using 1,1,3,3-tetraethoxypropane standard and based method of the reaction between thiobarbituric acid (TBA) and MDA. The absorbance rate of produced solution was determined spectrophotometrically (V-1201, Shimadzu, Japan) at 532 nm. The plasma lipid peroxidation (LP) values in terms of MDA were expressed as nmol/mL plasma [44,48].

### 2.4. Statistical Analysis

Data were analyzed using a completely randomized design as the factorial arrangement with R Software [49]. In order to assess normal and independent distribution, all random errors were homogenized of variance using the Shapiro–Wilk test and Bartlett’s test. Mean differences were compared using Duncan’s multiple range test [50] (Duncan, 1955), and values were expressed as means ± standard error of the means (SEM).

## 3. Results and Discussion

The characterization of bioactive phytochemicals in amaranth grains, shown in Table 3, shows the abundance of different types of phytosterols and tocopherols and, subsequently, the outstanding presence of squalene, which makes amaranth unique compared to other edible plants. The occurrence of such compounds in food sources indicates the value of superior nutrients, and at the same time, consumers can benefit from the potential benefits of this source to improve health and reduce oxidative stress. The presence of these bioactive compounds and their effects has been reported in other studies by Ogrodowska et al. [51], Tang and Tsao [12], Iftikhar and Khan [52], and Waisundara [53], demonstrating the beneficial effects and the high value of amaranth grain as food or feed.

The findings related to the productive performance of laying hens (Table 4) showed that the main effect of including a high level of RAG in diet determined a decrease in FI and an increase in FCR (*p* ˂ 0.05). Moreover, increasing RAG levels determined a significant decrease in HDP, EW, and EM (*p* ˂ 0.05), but the main effect of enzyme addition led to improved productive performance parameters (*p* ˂ 0.05). The interaction effects indicated that with increasing levels of RAG, the decrease in egg production was significant, but an improvement was observed with the addition of the enzyme blend (*p* ˂ 0.05). Feeding RAG up to 10% with enzyme blend supplementation led to similar performance to the control group. These results are consistent with the findings of Tillman and Waldroup [54], who reported that the use of amaranth grain at 10, 20, and 30% of the diet of laying hens led to a decrease in FI, but despite the refusal of feed in amaranth diets, no significant variations were observed in daily weight gain or the health status of hens; moreover, feed efficiency was also improved. The same researchers attributed the decrease in FI in birds fed more than 20% of amaranth to the presence of phenolic compounds and saponins in raw amaranth grain [55]. In addition, our results were in line with those of Popiela et al. [23], who reported that the consumption of amaranth grain at 5 and 10% of the diet did not significantly influence the FI of laying hens. It was observed by Pedersen et al. [56] that a high level of lignin in the amaranth cortex was negatively correlated with energy and digestibility, and decreased the bioavailability of nutrients. Decreased FI in laying hens fed high levels of amaranth in their diet could be attributed to increased dietary fiber and non-nutrients, decreased palatability, bitter matter, and less tendency of birds to consume diets [57]. This will increase the availability of important nutrients such as starch, protein, and minerals within the cell walls rich in crude fiber. The observed result of HDP reduction at high levels of amaranth was in contrast to the previous results of Tillman and Waldroup [54], who reported that levels of 10 and 20% of dietary amaranth led to increased HDP. Moreover, at a 30% inclusion level, the same researchers did not observe a decrease in egg production. Perhaps one of the reasons for this difference was the use of extruded processed amaranth grain. Popiela et al. [23] reported that a 5% level consumption of amaranth grain could improve HDP, which was inconsistent with the results of this study, but at 10%, the same researchers reported a decrease in HDP that was in line with our study. Additionally, the increase in EW and HDP reported by Tillman and Waldroup [54] could be attributed to the use of processed amaranth grain and to the presence of linoleic acid, which differed from the results of the present study. The decrease in egg production when fed more than 20% of processed amaranth with or without enzymes was observed recently by Hosseintabar-Ghasemabad et al. [40], who similarly used raw amaranth with or without enzymes. Many studies demonstrated that the efficacy of enzyme blends on laying-type poultry performance, as well as the improvement in EW and EM, was due to the fact that enzymes can lead to reduced concentrations of antinutritional factors and NSP [34,58], and viscosity [59] in edible plants such as amaranth. The use of enzyme blends also improved the activity of lipase and chymotrypsin enzymes in the gut, enhancing the digestibility of nutrients [60] and metabolizable energy [41]. It seems that this set of factors led to improved poultry performance [34,61].

The results shown in Table 5 indicate that at the end of the feeding period, egg quality traits, including shell thickness and strength, shape index, and Haugh unit, were not significantly affected by diets, considering both the main and interaction effects; however, with increasing consumption of RAG in diet, the main and interaction effects showed a significant decrease of 7 to 12% in yolk cholesterol compared to control (*p* ˂ 0.05). This was in line with the results of Punita and Chaturvedi [32] reporting that the use of raw or processed amaranth at 25% in hens’ diet can reduce yolk cholesterol. These researchers assessed that high amounts of fiber, as well as the presence of squalene in amaranth, led to increased hepatic 3-hydroxy-3-methylglutaryl coenzyme (HMG-CoA) reductase activity, which helps prevent cholesterol deposition in egg yolk. Furthermore, the presence of a high level of linoleic acid in amaranth grains led to the excretion of bile acids, lowering cholesterol levels. Many edible plants and even their by-products, including amaranth, due to the presence of fiber, phenolic compounds, as well as bioactive compounds, can also increase cholesterol-lowering effects without adverse effects on the performance and health status of poultry [36,40,44]. Reklewska et al. [62] reported a decrease in yolk cholesterol using amaranth in laying hen diets, which is in line with the present study. Conversely, Escudero et al. [63] argued that the protein fraction of amaranth grain contains a relatively high amount of fiber, which can lead to anti-cholesterol effects. Several mechanisms have been reported for the effect of amaranth on the reduction in yolk cholesterol. In general, the liver and ovary are the primary sites of cholesterol biosynthesis in laying hens, and the liver is the main site of fat synthesis in the egg yolk. Given that amaranth grains have been proven to be a rich source of squalene, tocopherols, and tocotrienols, they can play an important role in cholesterol biosynthesis. Squalene is an unsaturated triterpene containing 30 polyprenyl carbons; it is structurally similar to beta-carotene, and it is well known as an intermediate of cholesterol synthesis. Squalene and its precursors, including oxidosqualene and bis-oxidosqualene, can be the precursors of 200 triterpenes, which can play many important roles in the body. Squalene in feed can reach the liver with the help of chylomicrons and is used to make steroids and bile acids. Bile acids (colic and deoxycholic) are made from squalene-synthesized cholesterol in liver cells and can combine with glycine and taurine to produce bile salts which have hypocholesterolemic effects in the body. Lower egg cholesterol has a positive correlation with lower cholesterol in the blood and liver [64]. Therefore, any factor that lowers blood cholesterol can lead to a decrease in yolk cholesterol, seeing how a five percent inhibition of HMC-CoA reductase activity leads to a two percent decrease in blood serum cholesterol. Due to the rate of bile secretion in laying hens, which is about 1 mL per kg of body weight per hour, the process of lowering blood cholesterol due to increased turnover of bile acids led to a decrease in egg yolk cholesterol. Feeding of amaranth can also bind cholesterol to bile acids and increase the fermentation effect on the production of short-chain fatty acids (SCFA), preventing the formation of micelles [40]. The cholesterol-lowering potential of amaranth, reviewed by Peiretti [19], supported the hypothesis that this plant may be successfully used in livestock nutrition to produce high-quality products.

The effects of feeding RAG and enzyme blend on blood parameters of laying hens are reported in Table 6. The findings of the main effects of RAG showed no significant differences in hens’ blood triglyceride (TG) and LDL (*p* > 0.05); however, blood total cholesterol (TC) and atherogenic index in all groups fed RAG decreased significantly compared to the control diet. Moreover, blood HDL showed an increase with increasing levels of RAG in diet (*p* ˂ 0.05). The main effect of enzyme supplementation was effective on blood TC (*p* ˂ 0.05). The interaction effects showed that RAG without enzyme blend had determined a significant reduction in blood cholesterol at concentrations higher than 10% compared to the control group, whereas including the enzyme blend in the RAG diets led to the lowest blood cholesterol levels (*p* ˂ 0.05). In addition, regarding blood LDL, only the diet with 40% RAG without enzyme showed the lowest value (*p* ˂ 0.05). The results of interaction effects for blood HDL indicated that consuming RAG with or without enzyme blend led to higher HDL levels than the control group (*p* ˂ 0.05). The output of results of the mutual effects on atherogenic index showed that this index was able to show a significant and promising reduction in response to the addition of RAG with or without enzymes (*p* ˂ 0.05).

Previous studies showed that amaranth can play a key role in reducing blood cholesterol in both animals and humans due to the amounts of unsaturated fatty acids, the abundance of squalene, and the high amount of phytosterols and tocopherols it contains [65,66]. In a study, Qureshi et al. [27] reported that supplementing the diet of broilers with grains from two *Amaranthus* species (*Amaranthus hypochondriacus* and *Amaranthus cruentus)* led to a 10–30% of reduction in blood cholesterol. The LDL levels showed up to 40% of reduction, which was consistent with the results in blood cholesterol and LDL in the present study. The aforementioned researchers also demonstrated that the activation of 7-alpha hydroxylase enzyme led to the formation of bile acids from cholesterol and increased the catabolism and excretion of cholesterol. The authors assessed that the dietary feed source containing isoprenoids, while having squalene, tocotrienols, tocopherols, crude fiber, and saponin, can effectively reduce the activity of HMG-CoA enzyme, leading to a significant reduction in blood cholesterol in chickens fed amaranth grains. Lehmann et al. [67] confirmed that feeding of tocopherol and tocotrienols, gamma, and zeta tocotrienols identified in amaranth grain played a role in reducing blood cholesterol. Mendonça et al. [28] and Soares et al. [29] reported that consumption of amaranth grain can reduce cholesterol in hamster blood by 48%. They also stated that when squalene is provided through feed consumption, a certain amount is converted into cholesterol, but such synthesis does not lead to an increase in cholesterol levels. These researchers attributed the decrease in blood cholesterol of hamsters fed amaranth grain to the presence of peptides that limited the production of the HMG-CoA reductase enzyme. However, there are several reports assessing the role of squalene in reducing blood cholesterol [40]. In a study, Janevski et al. [68] observed that total and LDL cholesterol in mice consuming cholesterol-rich food decreased significantly after injection with 3.5 mg of squalene, due to changes in gene expression. Farvin et al. [69] demonstrated that dietary squalene can have beneficial effects on blood pressure and hypercholesterolemia by keeping cholesterol and lipoprotein levels at normal levels. Recently, Leukebandara et al. [70] observed a decrease in blood cholesterol, LDL, and triglycerides by feeding raw and heat-treated amaranth grain in lactating goats.

The presence of sufficient amounts of methionine in amaranth may increase the production of lecithin in the liver, also leading to a decrease in cholesterol and liver fat [71]. Moreover, it was demonstrated that the tocopherols in amaranth grain may potentially reduce the synthesis of cholesterol, LDL, and lipoprotein lipase enzyme, and finally regulate and reduce cholesterol [12,27,72,73,74]. In addition, the presence of specific phytochemical compounds in amaranth grain, such as 20-hydroxyacidisone (20HE), can lead to anti-obesity effects in terms of reduction in triglycerides, cholesterol, and LDL in mice [12,75]. An investigation conducted by Króliczewska et al. [76] on blood parameters in laying hens showed that supplementing up to 10% of diets with amaranth (*A*. *cruentus*) led to the significant reduction in blood lipid parameters, which was in agreement with the present study. Conversely, Popiela et al. [23] did not observe any reduction in cholesterol and other blood lipid parameters in laying hens fed with amaranth, and the reason was probably due to the use of low amaranth grain levels (5 and 10%). Furthermore, several reports have also demonstrated that the presence and role of carbohydrate enzymes (such as pectinase, cellulase, furyl-esterase, and glucanase) led to the release of phenolic compounds in amaranth grain, which can indirectly play an effective role in reducing cholesterol and in improving blood lipid parameters [12,30,77].

Investigation of laying hens’ blood antioxidant status, in terms of total antioxidant capacity (TAC), malondialdehyde (MDA), alanine aminotransferase (ALT), and aspartate aminotransferase (AST), indicated that the main effect of RAG and enzyme as well as their interaction exhibited no differences without any negative effect (*p* > 0.05). Considering the breeding of laying hens in a cage system and the possible stress conditions in this type of rearing system, there was a fluctuation in blood antioxidant status; therefore, the use of feed sources such as amaranth may improve the antioxidant capacity of birds. In a previous study, Longato et al. [78] reported a significant decrease in serum lipid peroxidation level by feeding *Amaranthus caudatus* grain to broiler chickens at 0, 5, and 10% of amaranth, which was in disagreement with the findings of the present study. Alvarez et al. [79] found that the presence of phytosterol bioactive compounds, tocopherols, and squalene had a significant role in anti-inflammatory and antioxidant effects, reducing the risks of oxidative stress and also improving the consumer’s health. Moreover, vitamin E homologs, having an antioxidant effect, are particularly concentrated in amaranth grains compared to other grains, which can positively regulate physiological and metabolic processes [12,80,81,82,83]. The experimental diets in the present study did not show any negative effects on the blood antioxidant status of laying hens.

## 4. Conclusions

According to the findings of the present feeding trial, the consumption of raw amaranth grain had no negative effect on egg production and quality, mainly due to the abundance of phytosterol and tocopherol bioactive compounds on raw amaranth grains, along with the presence of squalene, leading to an improvement in the health parameters of birds, in terms of their blood lipid profile and antioxidant status. Moreover, a significant reduction in egg yolk cholesterol by feeding amaranth may be considered a valuable option for consumers’ choices. Thus, the use of RAG up to 10% along with enzyme blend addition can be recommended as an acceptable practical proposal for the poultry industry.

## Figures and Tables

**Table 1:** Design of the experimental dietary treatments.

Treatment	Raw Amaranth Level (RAG, %)	Enzyme (E)	Factorial Method (RAG × E)	Summarized
T_1_	0	−	0 × 0	0 RAG ^−E^
T_2_	10	−	10 × 0	10 RAG ^−E^
T_3_	20	−	20 × 0	20 RAG ^−E^
T_4_	30	−	30 × 0	30 RAG ^−E^
T_5_	40	−	40 × 0	40 RAG ^−E^
T_6_	0	+	0 × 0.025	0 RAG ^+E^
T_7_	10	+	10 × 0.025	10 RAG ^+E^
T_8_	20	+	20 × 0.025	20 RAG ^+E^
T_9_	30	+	30 × 0.025	30 RAG ^+E^
T_10_	40	+	40 × 0.025	40 RAG ^+E^

**Table 2:** Ingredients and chemical composition of the diets fed to laying hens.

Treatments ^1^										
**Ingredient (%)**	**T_1_**	**T_2_**	**T_3_**	**T_4_**	**T_5_**	**T_6_**	**T_7_**	**T_8_**	**T_9_**	**T_10_**
Corn	61.49	54.79	48.15	41.56	32.67	61.24	54.48	47.89	41.30	32.81
Soybean meal	23.56	21.71	19.82	17.86	16.22	23.59	21.84	19.91	17.95	16.24
Raw amaranth grain	0	10.00	20.00	30.00	40.00	0	10.00	20.00	30.00	40.00
Oyster mineral	9.67	9.11	8.54	7.98	8.55	9.71	9.11	8.54	7.97	8.39
Vegetable oil	2.40	1.62	0.84	0.03	0.00	2.58	1.80	1.01	0.21	0.00
Dicalcium phosphate	1.88	1.88	1.88	1.87	1.86	1.88	1.88	1.88	1.87	1.86
Vitamin premix ^2^	0.25	0.25	0.25	0.25	0.25	0.25	0.25	0.25	0.25	0.25
Mineral premix ^3^	0.25	0.25	0.25	0.25	0.25	0.25	0.25	0.25	0.25	0.25
Salt	0.20	0.20	0.20	0.20	0.20	0.20	0.20	0.20	0.20	0.20
DL-Methionine	0.30	0.19	0.07	0.00	0.00	0.30	0.19	0.07	0.00	0.00
Natuzyme P_50_^3^ Enzyme	0.00	0.00	0.00	0.00	0.00	0.025	0.025	0.025	0.025	0.025
Calculated nutrient content (%)										
AME_n_ (kcal/kg)	2830	2830	2830	2830	2830	2830	2830	2830	2830	2830
Crude protein	15.25	15.25	15.25	15.25	15.25	15.25	15.25	15.25	15.25	15.25
Ether extract	5.02	4.64	4.25	3.85	4.14	5.18	4.80	4.41	3.87	4.14
Crude fiber	2.78	3.83	4.88	5.92	6.94	2.78	3.82	4.87	5.92	6.94
Linoleic acid	1.58	3.95	4.31	5.06	5.86	1.58	3.95	4.31	5.06	5.86
Ca	4.35	4.35	4.35	4.35	4.45	4.35	4.35	4.35	4.35	4.35
P available	0.46	0.46	0.46	0.46	0.46	0.46	0.46	0.46	0.46	0.46
Meth	0.37	0.41	0.42	0.42	0.42	0.37	0.41	0.42	0.42	0.42
Meth + Cyst	0.67	0.67	0.67	0.72	0.72	0.67	0.67	0.67	0.72	0.72
Lys	0.78	0.79	0.81	0.81	0.82	0.78	0.79	0.81	0.81	0.82
Arg	0.81	0.82	0.82	0.85	0.86	0.81	0.82	0.82	0.85	0.86
Thr	0.57	0.58	0.59	0.61	0.61	0.57	0.58	0.59	0.61	0.61

^1^ Treatments: T1: %0 RAG-E, T2: %10 RAG-E, T3: %20 RAG-E, T4: %30 RAG-E, T5: % 0 RAG-E, T6: %0 RAG+E, T7: %10 RAG+E, T8: %20 RAG+E, T9: %30 RAG-E, T10: %40 RAG+E. ^2^ Vitamin supplement provided per kg of diet: vitamin A, 8000 IU; vitamin E, 20 IU; menadione, 3.0 mg; vitamin D3, 2000 IU; riboflavin, 4.0mg; capantothenate, 12 mg; nicotinic acid, 50 mg; choline 300 mg; vitamin B12, 15 mg; vitamin B6, 0.12 mg; thiamine, 1.5 mg; folic acid, 1.00 mg; biotin, 0.10 mg. ^3^ Mineral supplements provided per kg of diet: trace minerals (mg/kg of diet): Mn, 100; Zn, 70; Fe 50; Cu 10; Iodine 1; Se, 0.30; Antioxidant 50.00. SEM: standard error of the mean.

**Table 3:** Bioactive phytochemical compounds of raw amaranth grains.

Item	Value
Total phytosterols (mg/kg)	3254.28
β-Sitosterol (%)	38.49
Δ-5-Avena sterol (%)	27.32
Stigmasterol (%)	19.56
Total tocopherols (ppm)	550.86
α-Tocopherol (ppm)	18.61
β- and γ-Tocopherols (ppm)	293.18
Δ-Tocopherol (ppm)	239.07
Squalene (ppm)	2173.40

**Table 4:** Effect of experimental diets on productive performance of laying hens.

Item	Feed Intake (g/day)	HDP (%)	EW (g)	EM (g/day)	FCR (kg feed:kg egg)
Raw Amaranth Grain (RAG, %)					
0	109.17 ^b^	81.80 ^a^	60.56 ^a^	49.54 ^a^	2.20 ^d^
10	109.47 ^a^	80.35 ^b^	59.05 ^b^	47.46 ^b^	2.31 ^c^
20	109.43 ^ab^	79.52 ^c^	56.65 ^c^	45.05 ^c^	2.43 ^b^
30	108.75 ^c^	78.61 ^d^	55.51 ^d^	43.64 ^d^	2.49 ^a^
40	108.86 ^c^	77.64 ^e^	56.20 ^cd^	43.63 ^d^	2.49 ^a^
SEM	0.091	0.255	0.333	0.273	0.014
Enzyme (E, %)					
0 (^−E^)	109.03 ^b^	79.46	56.76 ^b^	44.92 ^b^	2.42 ^a^
0.025 (^+E^)	109.25 ^a^	79.71	58.42 ^a^	46.81 ^a^	2.35 ^b^
SEM	0.057	0.161	0.211	0.173	0.008
RAG × E					
0 × 0	109.03 ^cd^	81.45 ^ab^	60.20 ^a^	49.04 ^a^	2.23 ^de^
10 × 0	109.27 ^bc^	79.91 ^cd^	57.55 ^b^	45.99 ^b^	2.34 ^c^
20 × 0	109.64 ^ab^	79.01^def^	55.77 ^cd^	44.07 ^c^	2.45 ^b^
30 × 0	108.44 ^e^	77.98 ^fg^	54.75 ^d^	42.69 ^d^	2.54 ^a^
40 × 0	108.75 ^de^	77.05 ^g^	55.56 ^cd^	42.81 ^d^	2.55 ^a^
0 × 0.025	109.31 ^abc^	82.14 ^a^	60.91 ^a^	50.03 ^a^	2.18 ^e^
10 × 0.025	109.67 ^a^	80.80 ^bc^	60.55 ^a^	48.92 ^a^	2.26 ^d^
20 × 0.025	109.22 ^c^	80.02 ^cd^	57.54 ^b^	46.04 ^d^	2.40 ^bc^
30 × 0.025	109.06 ^cd^	79.24 ^de^	56.27 ^bc^	44.58 ^c^	2.44 ^b^
40 × 0.025	108.97 ^cd^	78.23 ^ef^	56.84 ^bc^	44.46 ^c^	2.45 ^b^
SEM	0.129	0.361	0.471	0.387	0.019
*p*-value					
RAG	0.001	0.001	0.001	0.001	0.001
E	0.011	0.291	0.001	0.001	0.001
RAG × E	0.005	0.005	0.001	0.001	0.003

^a–g^ Means within each column with different superscripts differ significantly at (*p* ˂ 0.05). HDP: hen day production; EW: egg weight; EM: egg mass; FCR: feed conversion ratio. SEM: standard error of the means.

**Table 5:** Effect of experimental diets on egg quality traits and yolk cholesterol of laying hens.

Item	Shell Thickness (mm)	Shell Strength (kg/cm^2^)	Shape Index (%)	Egg Specific (g/cm^3^)	Haugh Unit	Yolk Cholesterol (mg/g)	Yolk Cholesterol (mg/egg)
Raw Amaranth Grain (RAG, %)							
0	0.306	3.323	74.50	1.083	79.66	12.59 ^a^	218.26 ^a^
10	0.307	3.310	74.39	1.080	79.84	11.66 ^b^	201.87 ^b^
20	0.302	3.293	74.35	1.078	80.32	11..39 ^b^	196.85 ^b^
30	0.305	3.330	74.26	1.080	80.30	11.27 ^b^	193.77 ^b^
40	0.303	3.333	74.80	1.082	80.15	11.24 ^b^	193.03 ^b^
SEM	0.005	0.032	0.694	0.001	1.011	0.235	4.010
Enzyme (E, %)							
0 ^(−E^)	0.304	3.314	74.79	1.080	79.72	11.69	201.22
0.025 (^+E^)	0.306	3.322	74.13	1.081	80.39	11.57	200.28
SEM	0.003	0.020	0.439	0.001	1.063	0.148	2.536
RAG × E							
0 × 0	0.307	3.317	73.64	1.083	79.73	12.77 ^a^	220.99 ^a^
10 × 0	0.305	3.305	73.96	1.081	79.5	11.72 ^bc^	201.92 ^bc^
20 × 0	0.300	3.287	74.43	1.077	79.74	11.42 ^bc^	196.64 ^c^
30 × 0	0.302	3.327	74.37	1.081	79.92	11.29 ^c^	193.74 ^c^
40 × 0	0.306	3.332	74.75	1.081	79.71	11.25 ^c^	192.84 ^c^
0 × 0.025	0.305	3.330	74.75	1.083	79.58	12.42 ^ab^	215.53 ^ab^
10 × 0.025	0.310	3.315	75.15	1.080	80.19	11.60 ^bc^	201.82 ^bc^
20 × 0.025	0.305	3.300	74.74	1.079	80.90	11.36 ^bc^	197.06 ^c^
30 × 0.025	0.307	3.332	74.10	1.080	80.69	11.25 ^c^	193.79 ^c^
40 × 0.025	0.302	3.335	75.23	1.083	80.58	11.24 ^c^	193.21 ^c^
SEM	0.008	0.046	0.982	0.002	1.430	0.332	5.672
*p*-value							
RAG	0.975	0.908	0.985	0.381	0.987	0.001	0.001
E	0.699	0.775	0.295	0.768	0.467	0.577	0.794
RAG × E	0.971	1.00	0.920	0.899	0.993	0.001	0.001

^a–c^ Means within each column with different superscripts differ significantly at (*p* ˂ 0.05). SEM: standard error of the means.

**Table 6:** Effect of experimental diets on blood biochemistry and antioxidant status parameters of laying hens.

Item	TG	TC	LDL	HDL	Atherogenic Index	ALT (U/l)	AST (U/l)	MDA (nmol/mL)	TAC (U/mL)
Raw Amaranth Grain (RAG, %)									
0	99.98	105.86 ^a^	97.8	40.38 ^c^	2.42 ^a^	5.30	215.63	5.29	6.20
10	94.99	102.34 ^b^	96.19	47.07 ^ab^	2.04 ^bc^	5.14	215.07	5.05	6.36
20	94.65	100.91 ^bc^	96.46	47.12 ^ab^	2.04 ^bc^	5.01	214.45	5.09	6.39
30	96.29	99.43 ^c^	96.42	46.77 ^b^	2.06 ^b^	4.88	213.54	4.72	6.38
40	93.37	99.90 ^bc^	95.93	48.59 ^a^	1.98 ^c^	4.83	213.82	4.94	6.37
SEM	2.051	0.821	0.66	0.562	0.028	0.182	1.196	0.204	0.088
Enzyme (E, %)									
0 (^−E^)	96.75	102.85 ^a^	96.68	46.12	2.11	5.11	214.95	4.98	6.33
0.025 (^+E^)	96.96	100.52 ^b^	96.44	45.85	2.11	4.96	214.05	5.06	6.35
SEM	1.297	0.019	0.417	0.355	0.017	0.115	0.756	0.129	0.056
RAG × E									
0 × 0	100.23	106.73 ^a^	98.55 ^a^	40.23 ^c^	2.45 ^a^	5.45	216.36	5.11	6.17
10 × 0	95.35	103.51 ^ab^	96.56 ^ab^	46.29 ^b^	2.08 ^b^	5.22	215.68	4.99	6.33
20 × 0	95.23	102.68 ^bc^	96.78 ^ab^	46.86 ^b^	2.06 ^b^	5.12	214.81	4.84	6.38
30 × 0	96.44	100.58 ^cd^	96.20 ^ab^	46.78 ^b^	2.05 ^b^	4.89	213.74	4.83	6.39
40 × 0	96.52	100.74 ^cd^	95.33 ^b^	50.45 ^a^	1.89 ^c^	4.88	214.16	5.11	6.37
0 × 0.025	99.73	104.98 ^ab^	97.05 ^ab^	40.53 ^c^	2.39 ^a^	5.15	214.89	5.47	6.23
10 × 0.025	94.63	101.17 ^cd^	95.82 ^ab^	47.86 ^b^	2.00 ^bc^	5.07	214.47	5.10	6.38
20 × 0.025	94.06	99.15 ^d^	96.14 ^ab^	47.39 ^b^	2.03 ^b^	4.9	214.09	5.34	6.41
30 × 0.025	96.15	98.27 ^d^	96.65 ^ab^	46.76 ^b^	2.07 ^b^	4.88	213.34	4.6	6.35
40 × 0.025	100.22	99.06 ^d^	96.52 ^ab^	46.73 ^b^	2.06 ^b^	4.79	213.48	4.77	6.38
SEM	2.900	1.161	0.933	0.795	0.040	0.258	1.691	0.289	0.125
*p*-value									
RAG	0.319	0.001	0.329	0.001	0.001	0.380	0.720	0.394	0.539
E	0.912	0.003	0.680	0.598	0.964	0.356	0.407	0.659	0.797
RAG × E	0.917	0.033	0.034	0.026	0.025	0.985	0.998	0.545	0.995

^a–c^ Means within each column with different superscripts differ significantly at (*p* ˂ 0.05). TG: triglycerides; TC: total cholesterol; LDL: low-density lipoproteins; HDL: high-density lipoproteins; ALT: alanine aminotransferase; AST: aspartate aminotransferase; MDA: malondialdehyde; TAC: total antioxidant capacity. SEM: standard error of the means.

## Data Availability

Data is contained within the article.
